# “You can’t just jump on a bike and go”: a qualitative study exploring parents’ perceptions of physical activity in children with type 1 diabetes

**DOI:** 10.1186/s12887-014-0313-4

**Published:** 2014-12-20

**Authors:** Helen Quirk, Holly Blake, Beatrice Dee, Cris Glazebrook

**Affiliations:** Institute of Mental Health, University of Nottingham, Jubilee Campus, Triumph Road, Nottingham, NG7 2TU UK; School of Health Sciences, University of Nottingham, A Floor, South Block Link, Queen’s Medical Centre, Nottingham, NG7 2HA UK; Medical School, Queen’s Medical Centre, University of Nottingham, Nottingham, NG7 2UH UK; Division of Psychiatry and Applied Psychology, Institute of Mental Health, University of Nottingham, Jubilee Campus, Triumph Road, Nottingham, NG7 2TU UK

**Keywords:** Children, Type 1 diabetes, Physical activity, Parent perceptions, Qualitative, Thematic analysis

## Abstract

**Background:**

Parents of children with Type 1 Diabetes Mellitus (T1DM) have an important role in supporting diabetes management behaviours and helping to maintain their child’s healthy lifestyle. Physical activity has known benefits for children with T1DM [Diabet Med 31: 1163-1173], but children with chronic health conditions typically have low levels of physical activity. Research is needed to build an understanding of the experience of physical activity for children with T1DM. The purpose of this study was to understand parents’ perceptions of what influences physical activity for children with T1DM and to inform the practice of those working with children who have T1DM.

**Methods:**

Data were collected through semi-structured interviews with 20 parents (18 mothers, 2 fathers) who had a child aged 7 – 13 years with T1DM in the UK. Interviews were recorded, transcribed verbatim and data were analysed using thematic analysis [Qual Res Psychol 3: 77-101, 2006]).

**Results:**

Factors believed to influence participation in physical activity are presented as 7 major themes and 15 subthemes. Themes that emerged included the conflict between planning and spontaneous activity, struggles to control blood glucose, recognition of the importance of physical activity, the determination of parents, children relying on their parents to manage physical activity, the importance of a good support system and individual factors about the children that influence physical activity participation.

**Conclusions:**

This study highlights that parents serve as gate-keepers for children’s physical activity. The findings provide insight into the need for T1DM knowledge and competence in personnel involved in the supervision of children’s physical activities. Healthcare providers should collaborate with families to ensure understanding of how to manage physical activity. The findings sensitise professionals to the issues confronted by children with T1DM and their parents, as well as the methods used by children and their families to overcome obstacles to physical activity. The implications for further research, clinical practice, and physical activity promotion with children with T1DM are discussed.

**Electronic supplementary material:**

The online version of this article (doi:10.1186/s12887-014-0313-4) contains supplementary material, which is available to authorized users.

## Background

Type 1 Diabetes Mellitus (T1DM) is an autoimmune disease that permanently destroys beta cells in the pancreas. Insulin is not produced and blood glucose levels need to be carefully controlled [1]. Management of T1DM includes blood glucose monitoring, daily insulin injections, carbohydrate counting and regular physical activity [2]. In the UK, children with T1DM are advised to meet the recommended guidelines of at least 60 minutes of moderate-to-vigorous physical activity every day [3]. Some research has highlighted the benefits of regular physical activity for children with T1DM [4], yet other evidence suggests that children are insufficiently active to achieve the health benefits [5]. For example, Trigona et al. reported that 35% of Swiss children aged 6–17 years with T1DM reached the recommended 60 minutes of moderate-to-vigorous physical activity for their child per day versus 57% of children without T1DM [6]. To appreciate why some children with T1DM are inadequately active, research is needed to build an understanding of the experience of physical activity for children with T1DM.

Parents of children with T1DM have been known to take a dominant role in T1DM management until the child is at least 10 years of age, when parent and child enter a transition from parental management to child self-management [7]. Parents’ perceptions of their children’s T1DM may influence their decisions about parenting, which may have an effect on T1DM management behaviours and future health outcomes such as glycaemic control [8]. Therefore, parents are providers of, or gatekeepers to their child’s physical activity experiences [9], which warrants research exploring parents’ perceptions of their children’s physical activity.

Bandura’s Social Cognitive Theory (SCT) suggests that the environment and the individual affect one another in a process of reciprocal determinism to bring about any given behaviour [10]. Bandura suggests that children establish patterns of normative behaviour through role models encountered in their environment and everyday interactions [10]. As such, parents can influence children’s physical activity through modelling attitudes and providing encouragement [11,12]. Parents can also impact on children’s self-efficacy by providing rewarding opportunities to participate in physical activity and through encouragement and reinforcement [11]. Parents’ perceptions of what influences levels of physical activity can be used to inform the design of initiatives aimed at the promotion of physical activity in children with T1DM.

Opportunities for children’s participation in physical activity may be shaped by parental concerns and attitudes, for example, concerns over safety [13]. Falling blood glucose level (hypoglycaemia) is a common side-effect of physical activity in children with T1DM [14]. During the night, nocturnal hypoglycaemia can be common (approximately twice per month) and prolonged (up to 2 hours) in children with T1DM, especially on days of increased physical activity [14,15]. Parents of children with T1DM report anxiety and fear associated with hypoglycaemia [16]. Research has suggested that avoidance of hypoglycaemia is a high priority for parents of children with T1DM partaking in physical activity [17]. However, little is known about parents’ views of physical activity for their children with T1DM. Based on the idea that parental concerns and attitudes can shape children’s physical activity experiences, this study will explore parents’ perceptions of physical activity for their child.

To our knowledge, only one previous study has used qualitative methodology explored parental beliefs about physical activity in children with chronic conditions (T1DM, asthma and cystic fibrosis). Fereday and colleagues conducted focus groups with 25 Australian parents of children aged 4–16 years, 14 of whom had a diagnosis of T1DM [17]. Parental encouragement and parental attitude towards physical activity emerged as important motivators for children’s physical activity, which supports research findings from children without chronic conditions [18,19]. However, in Fereday et al.’s study, some aspects of parental support, particularly parental vigilance in planning and involvement in the activity, were unique to parents of children who had T1DM. These were believed to be instrumental in allowing the children to overcome perceived barriers to physical activity such as hypoglycaemia. Fereday et al. attributed parental vigilance to the investment time and money to supervise their child’s physical activity (e.g., thorough planning, driving long distances and providing equipment). This research is commended for providing an insight into how parents who have a child with a chronic illness perceive physical activity. Further research to explore parental beliefs about physical activity for children with T1DM is warranted. Qualitative methodology would achieve rich insights into this unique perspective.

In summary, it is believed that parents play an important role as facilitators of their child’s physical activity and parents of children with T1DM have specific diabetes-related concerns. The purpose of this study is to understand parents' perceptions of what influences physical activity for their children with T1DM and to inform the practice of those working with children who have T1DM.

## Methods

The study employed qualitative research methods, collecting data through in-depth semi-structured interviews. This study was informed by interrelated concepts of interpretivism and reflexivity balanced with pragmatism and transparency. This was achieved by seeking to understand the experiences and perceptions of parents whilst demonstrating practical implications for those working with children who have T1DM. The research was reviewed and approved by the University of Nottingham Medical School Research Ethics Committee.

Parents were recruited between February 2013 and March 2014 using a purposeful sampling approach with snowball techniques [20]. Parents who had a child aged 7–13 years with a clinical diagnosis of T1DM for at least three months were eligible. This age range was targeted because it encapsulates the preceding and early years of the period of transition between parental management and child self-management. An advertisement was placed in the Diabetes UK “Balance” magazine and parents responding to the advert were invited to contact the lead researcher (HQ) directly for more information. In addition, T1DM parent support groups across the UK were identified using an online search engine and support group leaders were emailed with request for an e-flyer to be distributed amongst group members. The e-flyer advertised the research and contained a link to a webpage (Bristol Online Surveys [21]) where parents could read about the study and provide their contact details confidentially. Those who supplied contact details were contacted by the lead researcher (HQ) via email who confirmed the parents’ eligibility and provided eligible parents with the study information sheet and consent form.

Parents agreeing to participate were scheduled for an interview. Participants were given the choice of a telephone interview or, where geographically feasible, a face–to-face interview. Face-to-face interviews took place in the participant’s home. Interviews were conducted at a mutually convenient time by a researcher; HQ (n = 16) or BD (n = 4). The interviewer received consent from the participant in writing (if interviewed in person) or verbally (if interviewed via telephone) prior to the interview. Both interviewers were aged between 20 and 25 years, were female, and were trained in qualitative methods and interview techniques. HQ was a PhD researcher and BD was a medical student. With the participant’s consent, interviews were recorded using an Olympus Dictaphone.

The interviewers aimed to create a free-flowing discussion directed by the interviewee in an informal conversational style. The questions used were open-ended and to ensure that same topics were covered across all interviews an interview guide was used (See Additional file 1 for Interview Guide). The interview guide sought to explore parents’ beliefs around physical activity with questions such as:

- What sorts of physical activities does your child take part in?
- What helps your child to be physically active?
- What makes physical activity more difficult for your child?

Interview length ranged from 15 to 120 minutes and the mean duration of interview was 50 minutes. Parents were offered a gift voucher after the interview.

Recruitment was guided by the number of participants needed to achieve theoretical data saturation [22]. According to Strauss and Corbin [22], theoretical data saturation refers to the point when i) no new or relevant data seem to emerge regarding a theme, ii) the theme is well developed and demonstrates variation, and iii) the relationships among themes are well established. Based on these criteria, the lead researcher (HQ) judged whether any new data were emerging that would inform our understanding of parents’ perceptions of physical activity or have practical implications for people working with children who have T1DM. No new data emerged (and therefore data saturation was achieved) at 20 participants, at which point recruitment ceased.

Almost all parents interviewed were mothers (18/20), two were fathers, eighteen were married, and the majority (17/20) had two or more children. The mean age of the child about whom the parent responded was 10.8 years (±2.2, range 7–14 years) and the mean length of diagnosis of T1DM was 4.7 years (±2.6, range 1–9 years). The study recruited parents of children between the ages of 7 and 13 years, however one parent’s daughter had turned 14 years-of-age when the interview took place and was included based on the belief that responses would not be substantively different from those of the target age range. Eighteen interviews were via telephone and two were face-to-face.

Audio recordings of the interviews were transcribed verbatim by the researcher who conducted the interview into Microsoft Word (Microsoft, Redmond, WA, USA), which enabled early familiarisation with the data. Participant anonymity was maintained by allocating participants an identification number and using pseudonyms for participants’ names within interview transcripts. Data analysis was an iterative process using a method of thematic analysis [23]. Thematic analysis is a method compatible with many different ontological and epistemological viewpoints.

Thematic analysis involved identifying codes, themes and common threads across all interview transcripts [23]. Six phases of thematic analysis based on Braun and Clarke (2006) were used. Phase 1; *familiarisation with the data*, involved transcribing, reading and annotating interview transcripts. Phase 2; *generation of initial codes*, involved listing ideas about what was interesting about the data and organising data into meaningful groups to form codes. Phase 3; *search for themes*, involved sorting codes into meaningful groups to form potential sub-themes. Phase 4; *review potential themes*, involved refining potential themes, ensuring codes within themes cohered together and ensuring clear distinction between themes. Related sub-themes were grouped and labelled with an overarching theme name. Phase 5 involved *defining and naming themes in a codebook*. In Phase 6; *final analysis and write-up*, findings were written up with verbatim extracts to demonstrate themes. This was not a rigid linear process, but an iterative process, moving backwards and forwards through the six phases as required. Codes were meaningful groups of data that captured the essence of data and could be events (e.g. hypoglycaemia: night-time), emotions (e.g. hypoglycaemia: parental concern) or beliefs (e.g. hypoglycaemia: very challenging). Codes could also refer to behaviours, values and attitudes. The software package NVivo version 10 (Qualitative Solutions and Research International) was used to facilitate the organisation of codes and themes, and has been used previously in similar research [13].

Codes were derived primarily from the data (inductive) but could also be theory-derived (deductive) [23]. Codes arose through a deductive approach when the theoretical understanding found in literature review allowed the researcher to be sensitive to certain topics that may arise in the data [22]. Examples of a priori codes were the hypoglycaemia codes stated above, as previous research has suggested that hypoglycaemia could be a common side-effect of physical activity and cause of concern for parents. Inductive codes were induced from data and thus not anticipated in advance of data analysis. Data analysis began with an inductive approach to ensure important aspects of the data were not missed. Deductive codes relating to specific areas of interest were then looked for in the data, but analysis was iterative rather than a rigid linear process.

Several approaches were used throughout the study to ensure methodological trustworthiness [24]. The researchers showed sensitivity, commitment and rigour (to theory, participants and data), transparency (e.g. being explicit with research decisions) and sought findings that would have practical implications. This was in addition to utilising a rigorous approach to establish the consistency and replicability of the themes [25].

To counter bias and enhance the credibility of the data, consistency of themes was explored. In accordance with the recommendations of Boyatzis [25], a codebook was developed which included a brief background to the study, a label for each theme, a theme and subtheme description and examples extracts that did and did not illustrate each theme [25]. Quotes belonging to each theme were selected at random and given to a second coder to code using the codebook. Boyatzis recommends that percentage agreement between two coders above 70% demonstrates acceptable reliability [25]. The percentage agreement between the two coders was established at 78%, indicating that the themes were consistent and reliable to a recommended standard [25]. This process resulted in minor amendments to descriptions of codes in the codebook, reassignment of some extracts to more appropriate themes and merging of two similar subthemes. Once the codebook was clarified, all previously coded transcripts were reviewed to ensure they were consistent with the revisions.

Reflexivity refers to the process of critically reflecting on the knowledge produced during the research process and the researcher’s role in producing that knowledge [23]. This was done explicitly by writing notes in a diary. Personal reflexivity entailed the researcher being careful to acknowledge personal background (e.g. *“I am a white, middle-class woman with a background in Psychology”)* biases (e.g. “*I have never experienced parenthood*”) and values (e.g. *“regular exercise is important to me”*) prior to and during the research process. During data collection, the researcher made notes about the interview, including impressions of the interview (e.g*. “I think Diane and I engaged very well”*), participant (e.g. *“It was apparent from the beginning that Diane was a very strong character and a passionate and determined mother of her children”*) and emerging points of interest (e.g. *“I was struck by Diane’s determination”*). In the early stages of data analysis, the researcher noted impressions, ideas and early interpretations of the data. This aided the generation of themes and served as a means for documenting the rationale for any changes or reassignment of codes and themes.

## Results

The purpose of this study was to understand parents’ perceptions of what influences physical activity for children with T1DM and to inform the practice of those working with children who have T1DM. Factors believed to influence participation in physical activity among children with T1DM are presented as 7 major themes and 15 corresponding subthemes. Themes are supported by verbatim quotes from parents.

### Theme 1 Conflict between careful planning and spontaneous activity

Parents perceived diligent planning and preparation to be fundamental to their child’s participation in physical activity, which conflicted with the spontaneous nature of children’s physical activity.

#### Parents recognise the importance of having a predictable routine

Parents in this study believed that planning and preparation enabled their child to participate in physical activity. Parents referred to everyday routines and also formal diabetes care plans, “I write down every day what he has to do that’s different, like today for P.E. [*physical education*] at what levels he can exercise at and what levels he can’t exercise at” (P02). When explaining what makes physical activity more difficult for children with T1DM, planning was mentioned e.g., “it’s a lot of effort and you’ve got to make sure you’ve got everything, and take extra stuff and you know, it’s not fun to be perfectly honest with you” (P16). When carefully prepared plans formed part of a routine, parents alluded to the predictability being facilitative, “he will do everything because it’s routine and he knows what to do and it’s well-practiced and rehearsed” (P11), whilst a disruption to routine was challenging for parents e.g., “We do have struggles every now and again, particularly when something different is happening because obviously it’s a change to routine…School trips spring to mind, sports day, fun days, swimming is a challenge” (P01).

#### Parents perceive problems with the spontaneous nature of children’s physical activity

The importance of routine and the vigilant planning for physical activity conflicted with the unpredictable nature of physical activity. For example, the title of this paper was taken from a quote that captured a viewpoint shared by many of the parents interviewed, “You can’t just jump on a bike and go, you have to think about how far you're going, what equipment you’ve got with you, has he tested beforehand, what levels he’s at” (P02). Unpredictability was often in reference to children’s spontaneous play, but some parents found structured activity sessions, such as training for a sports team, difficult to manage. This would be due to parents not knowing in advance what the training schedule would be and thus unable to anticipate what effect the activity would have on their child’s blood glucose level. For example, one mother explained why sometimes her daughter’s rowing training was difficult to manage, “you don’t know whether they’re going to do a hard racing session or whether they’re going to do a short one or whether it’s going to be lazy or work on technique, or she’s gonna go to the gym” (P08).

### Theme 2 Parents battle for blood glucose control

Parents perceived difficulty maintaining control of their child’s blood glucose levels during periods of physical activity, described by one father as a “constant battle” (P05).

#### Blood glucose monitoring requires vigilance and commitment from parents

Parents described their continuous commitment to blood glucose monitoring, which included numerous blood glucose tests before, during and after activity and throughout the day in order to control blood glucose levels. The arduous nature of this task for parents was demonstrated through references to it being a “24/7 job” (P08, P10) and a “constant balancing act” (P20). This could disrupt physical activity by delaying it e.g., “occasionally he has to join the [*activity*] class late because he’s too low or too high” (P02) or interrupting it e.g., “I had to make her get out of the pool half way through the lesson and dry off her finger and do a blood sugar, and that was quite awkward” (P13).

Attempts to manage blood glucose levels and physical activity were sometimes characterised by the method of trial and error, as summarised by one mother, “sometimes you get it right, sometimes you don’t [*laughs*]. Sometimes he comes home and he’s way too high because you’ve cut off too much, other times, you know, you’ve not cut off enough and he goes hypo” (P01). Synonymous with the nature of trial and error, parents described how attempts to manage their child’s physical activity can be unsuccessful e.g., “you can only really make your best guess based on previous experience and it still sometimes goes wrong” (P20) and difficult e.g., “they do say when you exercise then you get better blood sugars, but I don’t know, it just makes it more uncontrollable in some ways!” (P08).

#### Hypoglycaemia is challenging and a cause of concern for parents

Parents were aware that physical activity came with the risk of hypoglycaemia and conveyed that this was challenging to manage. The challenges faced by parents involved: i) the physical effect of hypoglycaemia, e.g., “he’ll just drop on the floor and become delirious” (P02) or having to stop participation e.g., “mid-way through a very impressive Frisbee session on Sunday morning when he pulled a spectacular hypo he had to come out for fifteen minutes” (P01); ii) the emotional impact of hypoglycaemia, such as frustration when hypoglycaemia impedes physical activity e.g., “he had a low and he missed break, and it’s devastating” (P01), or a lasting emotional impact of having a hypoglycaemic episode e.g., “[*the hypoglycaemic episode*] then coloured her whole view that she didn’t want to go into the P.E. lesson, to the point where she’d say she didn’t feel very well on those days she’s got P.E.” (P07); and iii) worry about hypoglycaemia e.g., “if she has a big hypo and she needs that extra assistance from outside and there’s nobody there that knows what to do, is always the worry” (P08). For some parents, the worry was more prominent earlier on in the diagnosis of T1DM, as one mother described her initial worries about skiing, “We weren’t too sure how a lot of activity, quite sustained activity for a couple of hours would affect her, so that was a worry I suppose at the time, but as time went on you know, we were able to learn and understand” (P17). However, another mother’s worry had “got harder over the years” (P13) because “the more parents I meet and talk to about checking in the night and stories that I hear about checks, I feel that I have to check her more often” (P13). Parental worry about nocturnal hypoglycaemia was coupled with more vigilant blood glucose monitoring in the evenings after activity and throughout the night.

It was not common for parents to talk about maladaptive hypoglycaemia avoidance behaviours, but one mother who had been concerned about her child skiing did allude to such behaviour, “we probably chose to run her blood sugars high rather than low, take the view that we’ll sort them out at lunchtime or whatever, sort them out later” (P17). A small number of parents confided that the challenge of managing blood glucose fluctuations made it tempting to avoid physical activity e.g., “the effect it has on her blood sugars, it’s easier for me that she doesn’t want to do it” (P13).

### Theme 3 Parents recognise the importance of physical activity

Parents in this study recognised the importance of physical activity for its desirable effect on their child’s health or behaviour.

#### Parents believe that physical activity is important for their child

Parents attributed the importance of physical activity to its health benefits for people with and without T1DM. Those who believed physical activity was important for T1DM gave reasons such as: health and fitness e.g., “they’ve got to keep themselves fit and healthy” (P02); disease prevention e.g., “because she’s at higher risk of heart disease” (P17); and longevity e.g., “to live a long life” (P06). Parents did not perceive T1DM to be the only reason why their child should keep physically active, but some believed that T1DM did provide an incentive to encourage their child to be active e.g., “I think knowing that it’s helping him stay healthy with his diabetes, obviously that’s sort of what we take into account” (P09)*.*

#### Parents could see the positive effect of physical activity in their child’s health or behaviour

Some parents not only held beliefs about the importance of physical activity, but also described having observed benefits of physical activity in their child’s health or behaviour. The overt benefits described by parents were physiological or psychological. Physiological benefits included improved blood glucose control, e.g., “makes things easier to control” (P09), “the more sport he did that the less hypos he was having” (P19) and body composition e.g., “I just noticed his physique changing and I think slowly he’s getting a thinner waist and broader shoulders” (P19). Some parents noticed psychological benefits such as, giving the child space, energy or anger release e.g., “a way of getting out his anger” (P19) and developing knowledge e.g., “[*being active*] makes him have a better understanding of the relationship between food and exercise and insulin” (P11).

### Theme 4 Parents are determined to overcome hurdles to physical activity

Parents demonstrated assertiveness, resilience and forcefulness to ensure that their child could have a ‘normal’ life and take part in any physical activity.

#### Parents demonstrate assertiveness

Determination was evident in parents’ accounts of being forceful and direct, particularly when negotiating care plans and arrangements with external bodies such as school and extracurricular activity groups. For example, “I think they [*school*] got supportive when I told them they had to be” (P01). Examples of assertiveness involved being firm with requests (e.g., “I’m very direct, not wishy washy about it” (P06)), setting clear boundaries (e.g., “I do the training for them [*the teachers at school*], because I don’t trust anyone else to do it” (P16)), standing up for the child’s rights, (e.g., “I’m just determined that my child is going to be healthy in the long-term and I’m not prepared to settle for second best” (P07)) and expressing opinions or disagreeing with others or policies at a reasonable volume. For example, two parents had issued formal complaints when their child had been excluded from school activities such as swimming lessons or activity trips (e.g., “we did take them [*school*] to court and we did ring the disability related discrimination tribunal” (P18)). Assertive parents also tended to show resilience to overcome barriers to enable their child to be physically active, as summarised by one mother, “if there’s a barrier to it [*activity*], we find a way through it” (P06). One mother conceptualised these barriers as ‘hurdles’, “they [*children with T1DM*] just have some hurdles to get over to get active (P01)”.

#### Parents want their child to have as normal life as possible

The majority of parents interviewed were determined for their child to experience normality, which entailed going to “enormous effort” (P11) to help their child overcome barriers to physical activity, e.g., “it is a lot more work, I’ll say that, but you do what you have to do for your child to have as normal life as possible” (P20). Normality for some parents included a life without diabetes, for example, “we’ve always tried to think, if he didn’t have diabetes, would we let him go [*on activity/trip*], and if the answer is yes then we should still let him go and try not to let it stop him” (P09). For some parents, the desire for their child to experience a ‘normal’ life conflicted with safety concerns, e.g., “[*at rowing club*] I need to know that the people taking her out on the water know that she’s diabetic …Then it’s sort of an issue, does everybody have to know?…an issue of privacy as well, although it is for her safety” (P08).

### Theme 5 Parents perceive their child’s participation in physical activity as dependent on parental management and supervision

The parents believed that their child’s participation in physical activity was dependent on parental supervision.

#### Parents perceive difficulties allowing their child to achieve independence

Parents realised the need for allowing their child independence, but described children’s dependence on their parents made this more difficult, e.g., “let him grow up independently but at the same time make sure he’s safe, it’s a real challenge” (P01). One father described, “the biggest thing that you lose as a child with Type 1 Diabetes is independence and freedom” (P05). He attributed the loss of independence to the necessary safety precautions that needed to be in place when a child with diabetes was physically active away from parents, “if we were confident of his ability to deal with it himself, then yes potentially we wouldn’t necessarily have the restrictions on clubs or wouldn’t necessarily have to miss the occasional one because we [*parents*] couldn’t sit outside” (P05).

Parents were involved in making important choices about physical activity, including the decision about whether to take part or not, “we have to think, what his [*blood glucose*] levels are going to be before he starts, at what level we actually let him participate or not” (P02). As such, several parents believed that this necessitated their presence during the physical activity, including structured external activities such as school activity holidays. For example, one mother described a conflict between her son’s autonomy and his safety, “we want Harry to have that freedom, so we literally sit outside the door in the car. Harry knows we’re there and they [*Cub Scout leaders*] know we are there if there’s a problem” (P06). Whilst some parents were happy with this responsibility, one mother confided that accompanying her son to activities had become burdensome, e.g., “We go through this again and again and again with everything he does whether it’s like, he went to Beavers and Cubs and Scouts he did all that because we were there every flaming week with him, sitting there bored as hell, can’t leave him” (P16). Some parents believed that their presence during their child’s physical activity influenced the child’s confidence, e.g., “He likes me being there for a bit, you know, it gives him a bit of security and confidence” (P02).

#### Parents are reluctant to give others responsibility

Parental responsibilities over their child’s participation in physical activity was pressurised by a reluctance to give the responsibility of care to other people, such as school personnel, activity leaders and other family members, e.g., “I’d prefer if she didn’t want to do a lot of sporty things, because I’m not happy leaving her” (P13). Reasons for this reluctance included: the specialised knowledge required by the supervisor of the child, e.g., “it’s really hard for me to let anybody else take her to do anything…because you have to think about so many things like what she’s had to eat and what her blood sugar was before you started and how many sweets she’s had or how many glucose tablets or what other food she’s had” (P13); lack of skilled staff e.g., “for Taekwondo I’m always there, because his teacher and everything, they’re not trained in how to treat him if he suddenly has a hypo or comes hyper or his cannula comes out (P02); other’s negative perception of T1DM e.g., “they’ll be flippant and not take it serious” (P06); others not willing to accept responsibility e.g., “the people who took it would say oh no I’m not dealing with that you will have to stay and deal with it” (P16) and; negative past experience of giving others the responsibility e.g., “at holiday club…they didn’t test him before dinner…and I said why didn’t you do his tests, and they said ‘oh we were busy’”. It was evident that the lack of education, understanding and awareness around T1DM in others was a problem for parents, as summarised by one mother, “if Harry’s P.E. teacher had been trained on how to deal with asthma and diabetes and it’s part of their training, then surely me as a parent would feel more comfortable” (P06).

### Theme 6 Parents recognise the importance of support systems

Parents identified figures they perceived to be important sources of support for their child’s participation in physical activity. Key supportive figures were the family, hospital staff, school teachers and active role models.

#### Parents perceive themselves as important in supporting and encouraging their child’s participation in physical activity

When asked to describe what helps their child be physically active, the vast majority of parents referred to their own involvement and encouragement. Involvement entailed direct involvement, e.g., “I sit outside Beavers every Tuesday and I sit at tennis every week” (P05) and shared interactions, e.g., “the family example…we’re going to walk round the forest, going to go for a cycle ride” (P08). Encouragement referred to parental attitudes e.g., “I love sport…so I’ve always, you know, even when we first had kids we wanted to sort of encourage that” (P05) and verbal encouragement, e.g., “we’ve both said ‘you have to do something’” (P15). Logistic support, such as provision of equipment, transport and funding was also believed to be important contributor to an active lifestyle, e.g., “we support him by financing the football things and taking him to various places that he needs to go” (P09).

#### Parents value the support received from the hospital

Parents valued the support received from their child’s diabetes clinic, and were appreciative of medical staff providing individualised advice and guidance, e.g., “we’ve always been able to contact them [*the hospital*] when we’ve had specific activities going on, like if we’ve been out on a long hike or he’s done long exercise, then we can discuss which insulin to drop and how to alter the ratio of the food” (P12). Parents who described a positive experience of the support received from the clinic referred to the medical staff being: available e.g., “you can contact [*the nurse*] most of the time, even outside office hours you can get hold of her” (P09); helpful e.g., “[*they*] help you work out ways for yourself to manage it” (P17); and encouraging of the child’s participation in physical activity. For example, one mother described how the nurse had supported her son in maintaining his previously active lifestyle, “[*the nurse*] was good because she tried to get him back into the running, she was very encouraging and she really helped with that, giving him diaries of people that ran with diabetes and what helped and what doesn’t” (P14).

However, some parents perceived the support they had received from the clinic as unhelpful or unaccommodating of their needs. For example, one mother believed that her concerns about night-time blood glucose testing were not supported, “if I say when she does more activity I’ll be checking even more at night, they [*medical staff*] don’t think I need to check at all [*during the night*]. So they don’t really understand” (P13). Another mother expressed anger at her daughter not being offered the support that was available to children involved in higher-level sport, which resulted in her daughter’s discontinuation of netball:

*[the doctor] said that children who play sport at a certain level are given intensive programmes of how to manage their diabetes when they go and play sport…and I felt quite strongly that, although Joanne would never be playing netball for England, she was quite a nice little club player and she deserved as much help with managing her diabetes as these other kids* (P15).

One mother described how inadequate information and support from the clinic had led to her son discontinuing Taekwondo after diagnosis because,

“*we hadn’t been given the information to handle it properly or the information we were given about what to do didn’t work for him and he became embarrassed about having hypos and having to sit out, so he did elect to stop that in the early days just after diagnosis because there wasn't enough support and information*” (P20).

#### Parents value the support received from school

Parents perceived that support and encouragement from school personnel was an important influence on their child’s participation in physical activity. Supportive school practices included: being receptive to diabetes training and knowledge acquisition, e.g., “they’ve learnt to use the technology that we’ve given them and they have made every effort to try and fit in with what we require” (P07); providing the opportunity to be active (i.e., inclusivity) e.g., “he’s never not been allowed to be completely involved in anything and everything that’s going on” (P10); and facilitation of blood glucose testing in relation to physical activity, e.g., “the P.E. teacher is like, ‘Sam, check, make sure you’ve got enough energy to play this match’, so he’ll check himself” (P19).

Generally, parents were satisfied with the support their child had received from school, but many parents could recall specific occasions when schools had been less supportive e.g., “the teacher refused to deliver any care in relation to Harry’s diabetes…very scary, everyday leaving him, wondering if he’s having a hypo” (P06). Parents perceived a lack of support when teachers demonstrated a lack of T1DM awareness and competence e.g., “teachers lacking confidence in dealing with hypos…teachers are absolutely terrified of hypos in sporting activity and so they will not push or challenge him at all” (P11). One mother described a time when her daughter missed her entitlement to P.E. due to the cold temperature of the swimming pool and no physical activity was offered as an alternative, “she ends up doing extra handwriting which she isn’t very happy about because she really would like to be more involved, she does like swimming” (P07).

#### Parents perceive active role models as important for their child’s participation in physical activity

Parents believed that physically active significant others served as role models for their children. Parents gave examples of role models, which frequently involved active parents e.g., “Role models. I mean when they started karate when they were five, their Dad joined with them” (P11). Role models also included siblings and activity leaders, for example, “we found a martial arts instructor who is Type 1, so that’s a role model. I think just everybody around him being active” (P11). Peers were also cited as important role models for children, especially those who were physically active e.g., “he’s made friends with like-minded people and they play football at lunchtime” (P09).

### Theme 7 Individual factors that influence participation in physical activity

#### Parents attribute participation in physical activity to their child’s personal characteristics and preferences

Parents often attributed their child’s participation in physical activity to their personal characteristics and preferences. Several parents described their child as being or not being a 'sporty' type, referring to their child’s enjoyment, ability and preference for sporting endeavours e.g., “he’ll do anything, he loves P.E. at school and he’ll have a go at whatever they’re doing, it doesn’t matter what it is, he’ll enjoy it and have a go” (P09). Those parents who described their child as enjoying physical activity tended to emphasise that their child would not let T1DM stop them from being active, which was perceived as a positive influence e.g., “I think if she can go out and do those things without the diabetes getting in the way too much then that’s really promising, at least she’ll continue when she gets bigger” (P08).

When parents described their child’s lack of enjoyment of physical activity, they often described alternative preferences such as sedentary screen-based activity, e.g., “he likes his iPod, he likes his phone, he likes the telly [*television*], he likes the laptop, you know and I’ve tried lots of different things but can’t get him interested in anything long-term things like skate-boarding, kick-boxing, karate” (P04). One mother alluded to enjoyment being akin with ability, “it’s not enjoyable if you're not good at it” (P16). The same mother alluded to the idea that her son could use diabetes as an excuse not to be physically active, “he will make up excuses about a hypo and check his bloods and get out of doing it” (P16). Some parents were keen to point out that their child’s interest in physical activity was not attributed to them having T1DM, as one father explained, “he pretty much hated all them [*activities*] to start with, again this is part of just his make-up, nothing to do with diabetes” (P05).

## Discussion

The purpose of this research was to understand parents' perceptions of what may influence physical activity for their children with T1DM. Interviews indicated that parents serve as gate-keepers for children’s physical activity and perceive challenges relating to their child’s participation in physical activity, but value the supportive systems that enable their child to overcome these hurdles. Themes that emerged included the conflict between planning and spontaneous activity, struggles to control blood glucose, recognition of the importance of physical activity, the determination of parents, children relying on their parents to manage physical activity, the importance of a good support system and individual factors about the children that influence physical activity participation.

The findings demonstrate how parents value the importance of routine and perceive a conflict between carefully arranged diabetes management plans and the sporadic, unpredictable nature of children’s physical activity. Previous research with children who have chronic conditions has shown that having management plans as part of daily routine was perceived by their parents as fundamental to children having an active lifestyle [17]. Research exploring young people’s (aged 13–16) and parents’ perspectives on T1DM self-management and glycaemic control has suggested that implementation of structured daily routine gives parents a sense of being ‘in control’ of diabetes [26]. This suggests that guidance is needed to help parents to implement physical activity management plans within a daily routine, and thereby generate competence to respond to spontaneous activities.

Parents described the constant battle to achieve desirable blood glucose control and perceived the need for more vigilant blood glucose monitoring during periods of physical activity. This is a similar finding to previous research which examined the day-to-day experience of mothers with young children who have T1DM and identified that mothers use the management behaviour of constant vigilance to accomplish the daily management of their child’s diabetes [27]. Elsewhere, research has shown that parents who have children with chronic conditions feel the need for continual monitoring of their child’s health status in relation to sport and physical activity [17].

A trial and error system for managing blood glucose level seemed to be a useful learning method for parents, which has also been found to play an important part in decisions about children’s asthma management [28]. Trial and error could be advantageous because professional advice can be tested and adapted to fit with parents’ own understanding of their child’s diabetes. However, this depends on parents having the knowledge and confidence to implement new techniques and respond to different outcomes. Some parents may need ongoing advice and support when learning about their child’s blood glucose level responses to physical activity.

Parents expressed concerns about the possible adverse side-effects of physical activity and spoke specifically about hypoglycaemia and nocturnal hypoglycaemia. Hypoglycaemia is more likely to occur after periods of physical activity [14]. Previous research has found that parents commonly report fear of nocturnal hypoglycaemia[29], and the current findings suggest that parental concern about hypoglycaemia may be heightened after physical activity. This highlights the importance of ongoing education about how to manage the side-effects of physical activity and designing interventions to support parents in normalising the hypoglycaemic response to physical activity.

Despite hypoglycaemia being perceived as a negative side-effect of physical activity, parents had observed benefits in their children such as improved blood glucose control, body composition and knowledge about the body’s response to food, exercise and insulin which they attributed to physical activity participation. Observable benefits could serve to reinforce parental beliefs about the importance of physical activity for their children. Parents held beliefs about the importance of physical activity for everybody and specifically for children with T1DM for reasons such as protection against disease. This suggests that educational resources may benefit from using techniques to raise awareness in parents about the short and long-term benefits of physical activity for children with T1MD.

Parents wanted to optimise their child’s physical activity opportunities, demonstrating a resilience to overcome or persevere in the face of challenges. Parents preferred to identify challenges as ‘hurdles to get over’ rather than barriers to stop their child participating in an active lifestyle. Resilience characteristics have been explored in families with a child who has T1DM and highlight that not all families have the qualities or resources to overcome adversity [30]. Such families may require ongoing support and guidance to overcome hurdles to physical activity such as hypoglycaemia.

Parents acknowledged that physical activity participation could foster independence in their children, but found it difficult to reach a balance between promoting independence and ensuring their child’s safety. Children’s physical activity was believed to be dependent on parental management and supervision, fostering a reluctance to pass on responsibility of their child to other adults. This supports previous findings in parents of children with chronic conditions [17]. Research has suggested that the responsibility of parents with a child who has T1DM is amplified due to the child’s reliance on parents to make decisions about diabetes treatment and behaviour [31], highlighting the importance of identifying social networks that may provide parents with support that could reduce this burden of responsibility.

Parents valued the supportive systems that enable their child to overcome some of the challenges relating to physical activity. The influential social agents included parents, school personnel, diabetes clinic staff and peers. Consistent with previous research in children with [17] and without [9, 32] diabetes, parents acknowledged that their own involvement, encouragement and logistic support facilitated their child’s physical activity. Active peers were also perceived as important enablers of children’s participation in physical activity. The Social Cognitive Theory can explain the influence of active parents and peers on children’s physical activity [10]. Bandura suggests that children learn behaviours through role models and as such, parents and/or peers who endorse physically active behaviour or attitudes could promote physical activity in children [10]. The findings also go some way to support Bandura’s concept of self-efficacy, suggesting that a child’s confidence or belief in their ability to be physically active might be enhanced in the presence of parents. Hence, targeting key influential figures such as parents and peers is warranted if attempts to promote physical activity in children with T1DM are to be successful.

Parents valued the support they received from their child’s diabetes clinic, especially when the healthcare providers were deemed to be available, helpful and encouraging of physical activity. On occasions, healthcare providers were perceived as unsupportive due to a perceived lack of mutual understanding between the parent and medical staff or a lack of appropriate information provided, which had resulted in discontinuation of physical activity. This is consistent with findings that have shown positive relationships with healthcare providers are imperative for optimal management of T1DM in childhood [33]. This highlights the important position of the healthcare professional to offer advice and support for children’s prolonged participation in physical activity. Research with healthcare professionals would be useful to explore their perceived competency to promote regular physical activity in young patients with T1DM and identify any support needs for communicating physical activity guidance.

Having a supportive school environment where physical activity was encouraged and supervised by trained and attentive teachers was perceived as facilitative of children’s physical activity. Dissatisfaction with the support from school resulted from a perceived lack of teacher awareness of T1DM and competence in management techniques. The school environment has been identified as an important correlate of physical activity [34,35]. A literature review investigating the effects of T1DM on schooling, including teachers’ awareness of T1DM, suggested that teachers felt uninformed about T1DM, were unable or unwilling to offer support (e.g., could not recognise or properly treat hypoglycaemia) and students with T1DM and their parents were apprehensive about school personnel’s limited understanding of diabetes [36]. The current findings offer some support for this and reinforce the point that people working with children (e.g., teachers) must be educated about T1DM and be trained to manage a child with T1DM during physical activity.

Parents perceived children’s individual characteristics such as their preference for or enjoyment of physical activity as an important influence on their uptake and maintenance of physical activity. Children’s enjoyment of physical activity has been perceived by parents as an important facilitator of physical activity [37], implying that parents believe their children are intrinsically motivated to participate in physical activity. The current findings would suggest that parents of children with T1DM share this belief. Helping parents to facilitate their child’s intrinsic motivation for physical activity could be a successful approach to physical activity promotion in children with T1DM.

### Practical implications

The research provides an in-depth look at the specific challenges, hurdles and barriers that parents of children with T1DM may face when their child is participating in physical activity. The findings may be used to extrapolate practical implications for the teaching and caring of children with T1DM. The high consistency of themes support the credibility of the findings and the reflexivity process enhances its methodological rigour. However, the findings should be considered in light of several methodological issues. The majority of the parents interviewed were married and were mothers and so the findings may not reflect the experiences of single-parent families or fathers. Research has demonstrated a difference between the influence of mothers and fathers on their child’s physical activity [12], warranting further research into the paternal perspective. Furthermore, due to the self-selected recruitment and the nature of the research question, the study may have reached the more motivated parents who actively seek assistance and information about physical activity. Parents with little interest or involvement in their child’s physical activity may have been underrepresented. Nevertheless, the perceptions of parents with physically active children are valuable for demonstrating how barriers to physical activity can be overcome [38].

## Conclusions

This study demonstrates the potential influence of parents' perceptions on the activity level of children with T1DM. The findings provide insight into the need for T1DM knowledge and competence in personnel involved in the supervision of children’s physical activities. The diabetes healthcare team have an ongoing opportunity to promote active lifestyles. Healthcare providers should collaborate with families to ensure understanding of how to manage physical activity. This sample was somewhat homogenous and further research is needed to investigate the experiences of parents who are in a less supportive position and less informed about physical activity. The findings sensitise healthcare providers and school personnel to the issues confronted by children with T1DM and their parents, as well as the methods used by children and their families to overcome obstacles to physical activity.

## Additional file

Additional file 1:
**Interview schedule used to guide all interviews.**

## References

[1] **About Diabetes** [http://www.idf.org/about-diabetes]

[2] Kiess W, Kapellen T, Siebler T, Deutscher J, Raile K, Dost A, Meyer K, Nietzschmann U (1998). Practical aspects of managing preschool children with type 1 diabetes. Acta Paediatr.

[3] **Start active, stay active: a report on physical activity from the four home countries' Chief Medical Officers.** [http://www.dh.gov.uk/en/Publicationsandstatistics/Publications/PublicationsPolicyAndGuidance/DH_128209]

[4] Quirk H, Blake H, Tennyson R, Randell TL, Glazebrook C: **Physical activity interventions in children and young people with Type 1 diabetes mellitus: a systematic review with meta-analysis.** *Diabet Med* 2014, **31:**1163–1173. 10.1111/dme.12531 PMC4232875 24965376

[5] Liese AD, Ma X, Maahs DM, Trilk JL (2013). Physical activity, sedentary behaviors, physical fitness, and their relation to health outcomes in youth with type 1 and type 2 diabetes: a review of the epidemiologic literature. J Sport Health Sci.

[6] Trigona B, Aggoun Y, Maggio A, Martin XE, Marchand LM, Beghetti M, Farpour-Lambert NJ (2010). Preclinical noninvasive markers of atherosclerosis in children and adolescents with type 1 diabetes are influenced by physical activity. J Pediatr.

[7] King PS, Berg CA, Butner J, Butler JM, Wiebe DJ (2014). Longitudinal trajectories of parental involvement in Type 1 diabetes and adolescents’ adherence. Health Psychol.

[8] Pattison HM, Moledina S, Barrett T (2006). The relationship between parental perceptions of diabetes and glycaemic control. Arch Dis Child.

[9] Gustafson SL, Rhodes RE (2006). Parental correlates of physical activity in children and early adolescents. Sports Med.

[10] Bandura A (2001). Social cognitive theory: an agentic perspective. Annu Rev Psychol.

[11] Trost SG, Sallis JF, Pate RR, Freedson PS, Taylor WC, Dowda M (2003). Evaluating a model of parental influence on youth physical activity. Am J Prev Med.

[12] Määttä S, Ray C, Roos E (2014). Associations of parental influence and 10–11-year-old children’s physical activity: Are they mediated by children’s perceived competence and attraction to physical activity?. Scand J Public Health.

[13] Veitch J, Bagley S, Ball K, Salmon J (2006). Where do children usually play? A qualitative study of parents’ perceptions of influences on children's active free-play. Health Place.

[14] Juvenile Diabetes Research Foundation Continuous Glucose Monitoring Study Group (2010). Prolonged nocturnal hypoglycemia is common during 12 months of continuous glucose monitoring in children and adults with type 1 diabetes. Diabetes Care.

[15] Tsalikian E, Mauras N, Beck R, Tamborlane W, Janz K, Chase H, Wysocki T, Weinzimer S, Buckingham B, Kollman C (2005). Impact of exercise on overnight glycemic control in children with type 1 diabetes mellitus. J Pediatr.

[16] Barnard K, Thomas S, Royle P, Noyes K, Waugh N (2010). Fear of hypoglycaemia in parents of young children with type 1 diabetes: a systematic review. BMC Pediatr.

[17] Fereday J, MacDougall C, Spizzo M, Darbyshire P, Schiller W: **"There's nothing I can't do - I just put my mind to anything and I can do it": a qualitative analysis of how children with chronic disease and their parents account for and manage physical activity.** *BMC Pediatrics* 2009, **9:**1. 10.1186/1471-2431-9-1 PMC2636806 19117528

[18] Sallis JF, Prochaska JJ, Taylor WC (2000). A review of correlates of physical activity of children and adolescents. Med Sci Sports Exerc.

[19] Biddle SJ, Atkin AJ, Cavill N, Foster C (2011). Correlates of physical activity in youth: a review of quantitative systematic reviews. Int Rev Sport Exerc Psychol.

[20] Patton MQ: *Qualitative evaluation and research methods.* Thousand Oaks, CA, US: SAGE Publications, inc; 1990.

[21] **Bristol Online Surveys (BOS) Service** [www.survey.bristol.ac.uk]

[22] Strauss A, Corbin JM: *Basics of Qualitative Research: Techniques and Procedures for Developing Grounded Theory.* Thousand Oaks, CA, US: SAGE Publications; 1998.

[23] Braun V, Clarke V (2006). Using thematic analysis in psychology. Qual Res Psychol.

[24] Yardley L, Smith JA (2008). Demonstrating validity in qualitative psychology. Qualitative psychology: a practical guide to research methods.

[25] Boyatzis R: *Transforming qualitative information: Thematic analysis and code development.* Thousand Oaks, CA, US: SAGE Publications, Incorporated; 1998.

[26] Spencer JE, Cooper HC, Milton B (2013). The lived experiences of young people (13–16 years) with Type 1 diabetes mellitus and their parents – a qualitative phenomenological study. Diabet Med.

[27] Sullivan-Bolyai S, Deatrick J, Gruppuso P, Tamborlane W, Grey M (2003). Constant vigilance: Mothers' work parenting young children with type 1 diabetes. J Pediatr Nurs.

[28] Callery P, Milnes L, Verduyn C, Couriel J (2003). Qualitative study of young people's and parents' beliefs about childhood asthma. Br J Gen Pract.

[29] Patton SR, Dolan LM, Henry R, Powers SW (2007). Parental fear of hypoglycemia: young children treated with continuous subcutaneous insulin infusion. Pediatr Diabetes.

[30] Koegelenberg GJ: **Resilience characteristics of families with a child with type 1 diabetes.** *MA Thesis.* Stellenbosch: Stellenbosch University, 2013.

[31] Hackworth N, Matthews J, Burke K, Petrovic Z, Klein B, Northam E, Kyrios M, Chiechomski L, Cameron F (2013). Improving mental health of adolescents with type 1 diabetes: protocol for a randomized controlled trial of the nothing ventured nothing gained online adolescent and parenting support intervention. BMC Public Health.

[32] Salmon J, Timperio A, Telford A, Carver A, Crawford D (2005). Association of family environment with children's television viewing and with low level of physical activity. Obes Res.

[33] Herrman JW (2006). Children's and young adolescents' voices: perceptions of the costs and rewards of diabetes and its treatment. J Pediatr Nurs.

[34] Biddle SJ, Whitehead S, O'Donovan TM, Nevill ME (2005). Correlates of participation in physical activity for adolescent girls: a systematic review of recent literature. J Phys Act Health.

[35] Ferreira I, Van Der Horst K, Wendel‐Vos W, Kremers S, Van Lenthe F, Brug J (2007). Environmental correlates of physical activity in youth–a review and update. Obes Rev.

[36] Wodrich DL, Hasan K, Parent KB (2011). Type 1 diabetes mellitus and school: a review. Pediatr Diabetes.

[37] Bentley G, Goodred J, Jago R, Sebire S, Lucas P, Fox K, Stewart-Brown S, Turner K (2012). Parents' views on child physical activity and their implications for physical activity parenting interventions: a qualitative study. BMC Pediatr.

[38] Rees R, Kavanagh J, Harden A, Shepherd J, Brunton G, Oliver S, Oakley A (2006). Young people and physical activity: a systematic review matching their views to effective interventions. Health Educ Res.

