# Supplementary material for: “You can’t just jump on a bike and go”: a qualitative study exploring parents’ perceptions of physical activity in children with type 1 diabetes
Source: BMC Pediatr. 2014 Dec 20;14:313. doi: 10.1186/s12887-014-0313-4 (PMC4297408; doi:10.1186/s12887-014-0313-4)
Supplement: Additional file 1: — Interview schedule used to guide all interviews. [file 12887_2014_313_MOESM1_ESM.pdf]

Exploring physical activity participation in children with Type 1 Diabetes: perceptions of parents

Telephone Interview Guide: Parents of children with Type 1 diabetes

Aim: understand parent's perceptions of physical activity for their children with T1DM from an ecological approach

1. **Introduction**

- General introduction
  - Is it a convenient time to talk?
  - Can you hear me clearly?

- Personal introduction
  - PhD researcher and nature of the research

- Recap purpose of the study
  - I'd like to ask you about your child's physical activity
  - **IF INTERVIEWEE IS HAPPY FOR ME TO DO SO,** the interview will be recorded and then I will type it all up to use as data for my research.
  - If there's any questions you do not want to answer then that's ok and if at any time you want the interview to stop that's fine too.
  - I expect the interview to last about 30-45minutes, is that ok?

- If it's ok with you I will turn the recorder on now and everything we talk about from now on will be recorded.

Start recording

Obtain verbal consent to participate on tape

Before we begin:

- Have you received and read the information sheet?
- Have you read the consent form?
- Have you asked all the questions you want to ask about this study?
- Now that I have recapped the purpose of the study and you have read the consent form, are you happy to continue with the interview?

RECEIVE VERBAL CONSENT BEFORE CONTINUATION

1. **Background information**

I'll start by asking you about you some general questions about home-life and diabetes.

- Who is in the family home at the moment?
  - Prompt brothers/sisters
- How old is x ?
- When did x get diagnosed with diabetes?
  - How was that?
- How is x managing the diabetes now?
- How does x get on in school?

2. **Physical activity in children with type 1 diabetes**

Next I am going to ask you about x's participation in physical activity.

- What sorts of physical activities does x take part in?
  - Prompt in school
  - Prompt out of school
  - How does x feel about taking part in physical activities?
  - Prompt Is there anything x particularly enjoys?
  - Prompt Is there anything that x finds difficult? Why.

- How do you think X's diabetes affects his/her participation in physical activities?
  - Prompt What about strenuous activities such as team sports or running?
  - How do you feel about the amount of physical activity x has?
  - Prompt (if appropriate) why do feel x should be more active?

- What helps x to be physically active?
  - Prompt Peers, siblings, family, diabetes team, school/teachers, coaches – anybody else.
  - How do these people help/not help?
  - Prompt What would help x to be more active?
  - Prompt What stops/would stop x being active?
  - Can you foresee any changes in x's level of physical activity?

3. **Demographic information**

- Would you mind if I asked you some questions about yourself now?
  - What area of the country do you live?
  - Do you work outside the home?
    - Are you in paid employment?

4. **Closing questions**

- Is there anything you would like to add about x's level of physical activity?

5. **Remuneration**

We would like to offer you an Amazon voucher to thank you for your time.

- Could you provide your address so that I can post it out to you?

6. **Thank you for your time.**
   Is there anything you are concerned about or would like to ask me before we finish?

   (If necessary – signpost to external source of support)

CONFIRM NO QUESTIONS OR CONCERNS

END OF INTERVIEW

Stop recording.
